# Community Laboratory Testing for *Cryptosporidium*: Multicenter Study Retesting Public Health Surveillance Stool Samples Positive for *Cryptosporidium* by Rapid Cartridge Assay with Direct Fluorescent Antibody Testing

**DOI:** 10.1371/journal.pone.0169915

**Published:** 2017-01-13

**Authors:** Dawn M. Roellig, Jonathan S. Yoder, Susan Madison-Antenucci, Trisha J. Robinson, Tam T. Van, Sarah A. Collier, Dave Boxrud, Timothy Monson, Leigh Ann Bates, Anna J. Blackstock, Shari Shea, Kirsten Larson, Lihua Xiao, Michael Beach

**Affiliations:** 1 Waterborne Disease Prevention Branch, National Center for Emerging and Zoonotic Infectious Diseases, Centers for Disease Control and Prevention, Atlanta, Georgia, United States of America; 2 Parasitology Laboratory, Division of Infectious Disease, Wadsworth Center, New York State Department of Health, Albany, New York, United States of America; 3 Minnesota Department of Health, St. Paul, Minnesota, United States of America; 4 Communicable Disease Division, Wisconsin State Laboratory of Hygiene, Madison, Wisconsin, United States of America; 5 Kentucky Division of Laboratory Services, Frankfort, Kentucky, United States of America; 6 Biostatistics and Information Management Office, National Center for Emerging and Zoonotic Infectious Diseases, Centers for Disease Control and Prevention, Atlanta, Georgia, United States of America; 7 Association of Public Health Laboratories, Silver Spring, Maryland, United States of America; Aga Khan University Hospital Nairobi, KENYA

## Abstract

*Cryptosporidium* is a common cause of sporadic diarrheal disease and outbreaks in the United States. Increasingly, immunochromatography-based rapid cartridge assays (RCAs) are providing community laboratories with a quick cryptosporidiosis diagnostic method. In the current study, the Centers for Disease Control and Prevention (CDC), the Association of Public Health Laboratories (APHL), and four state health departments evaluated RCA-positive samples obtained during routine *Cryptosporidium* testing. All samples underwent “head to head” re-testing using both RCA and direct fluorescence assay (DFA). Community level results from three sites indicated that 54.4% (166/305) of Meridian ImmunoCard STAT! positives and 87.0% (67/77) of Remel Xpect positives were confirmed by DFA. When samples were retested by RCA at state laboratories and compared with DFA, 83.3% (155/186) of Meridian ImmunoCard STAT! positives and 95.2% (60/63) of Remel Xpect positives were confirmed. The percentage of confirmed community results varied by site: Minnesota, 39.0%; New York, 63.9%; and Wisconsin, 72.1%. The percentage of confirmed community results decreased with patient age; 12.5% of community positive tests could be confirmed by DFA for patients 60 years of age or older. The percentage of confirmed results did not differ significantly by sex, storage temperature, time between sample collection and testing, or season. Findings from this study demonstrate a lower confirmation rate of community RCA positives when compared to RCA positives identified at state laboratories. Elucidating the causes of decreased test performance in order to improve overall community laboratory performance of these tests is critical for understanding the epidemiology of cryptosporidiosis in the United States (US).

## Introduction

Cryptosporidiosis is a gastrointestinal disease caused by the protozoan parasite *Cryptosporidium*. In immunocompetent individuals, cryptosporidiosis is generally characterized by self-limited diarrhea, abdominal pain, weight loss, anorexia, fatigue, joint pain, vomiting, fever, and/or headache; a potentially deadly diarrheal illness characterizes infections in immunocompromised individuals [1–4]. In the United States, it is estimated that 748,000 annual cases of diarrheal illness linked to cryptosporidiosis occur from waterborne, foodborne, zoonotic, childcare, and nosocomial cases and outbreaks [5]. The majority of these cases and outbreaks are caused by two of the more than 20 *Cryptosporidium* species identified—*C*. *hominis* and *C*. *parvum* [6].

Cryptosporidiosis has been a nationally notifiable disease since the Council of State and Territorial Epidemiologists (CSTE) called for reporting in 1994 [3]. The most recent report in the United States found the 2010 incidence rate of cryptosporidiosis to be 2.9%, more than double the incidence reported in the first full year of national reporting in 1995 [3]. The cause of this increase is unknown. It may reflect true rises in exposure and transmission, advancements in diagnostic testing, increased reporting following the introduction of new therapeutic treatments and electronic outbreak reporting systems, or a combination of these factors [7–9].

In confirmed cases of cryptosporidiosis, the *Cryptosporidium* organisms, antigens, or DNA must be detected [10][11]. The current gold standard for testing is direct fluorescence assay (DFA) with a sensitivity of 93–100% and specificity of 99.8–100%[12–16], but a number of other methods are employed to detect *Cryptosporidium*, including acid-fast microscopy, enzyme immunoassay (EIA), indirect immunofluorescence assay (IFA), polymerase chain reaction (PCR), and rapid cartridge assay (RCA). Each assay has advantages and disadvantages relating to costs, resources (staff time and expertise, specialized equipment, consumables), turnaround time, sensitivity, specificity, and the ability to identify other pathogens. These factors must be considered to adopt the most appropriate method for a given population [17]. Cryptosporidiosis diagnosis in the absence of trained microscopists or molecular capabilities has been made possible with the introduction of RCAs that detect parasite antigen [18]. These commercially available RCA assays, such as Meridian Immunocard STAT! and Remel Xpect, work as qualitative immunochromatographic tests. *Cryptosporidium* antigen is detected using colored polystyrene particles or colloidal dye and labeled monoclonal antibodies to *Cryptosporidium*. When exposed to fecal specimens with *Cryptosporidium* antigen, a cartridge containing proprietary capture antibody for the antigen will cause a colored line (Remel Xpect, red for *Cryptosporidium*; Meridian Immunocard STAT!, gray-black) to appear in addition to a line in the control window. In previous studies, these assays were reported to perform well compared to other diagnostics assays [18–20].

However, as per a CSTE Position Statement, RCA results are excluded from cryptosporidiosis surveillance data, and cases with these laboratory tests are considered probable rather than confirmed [10]. This is following a Minnesota study in 2010 where RCAs were shown to have a low positive predictive value when specimens were subjected to confirmatory testing by PCR or DFA and modified Ziehl-Neelsen stain [21]. As more clinical laboratories implement these tests, accurate data on disease incidence in the US will be lost. Multiple factors may affect the accuracy and performance of RCAs, including the proficiency of the technologist conducting the test and reading the results, handling and storage of test kits, the appropriate use of sample preservatives, and storage times and temperatures [22]. In spite of these limiting factors, the ease-of-use and reduced labor costs of RCAs has resulted in their expanded use for the routine detection and reporting of cryptosporidiosis in the United States. The goal of the current study was to expand on the previous RCA findings and determine if RCAs can be used as reliable diagnostic tools for *Cryptosporidium* surveillance by “head to head” re-testing of RCA-positive *Cryptosporidium* stool specimens with both RCA and DFA.

## Materials and Methods

### Ethics statement

All case interviews were conducted as part of routine state surveillance activities. Personally identifiable information was not transferred to CDC as part of this study. All demographic and clinical data collected for this study were derived from existing surveillance data for cryptosporidiosis, a nationally notifiable disease in the United States. Thus, informed consent was conducted for each *Cryptosporidium* positive case as part of the state routine cryptosporidiosis surveillance and case investigation, based on the regulations of each site’s jurisdiction.

### Study sites

Four state health departments (Wisconsin, New York-Wadsworth, Minnesota, and Kentucky) were selected based on their responses to questions in a Request for Application distributed by APHL. Sites were asked to demonstrate access to at least 100 *Cryptosporidium* RCA-positive stool samples within one year, either as part of routine state submissions or through an established relationship with one or more community laboratory partners. Data from Kentucky were not included in analysis because <100 positive cryptosporidiosis cases occurred in Kentucky during the study period.

### Specimen collection

Stool specimens were collected during the study period (October 2012 through January 2014) from residual clinical samples that tested positive for *Cryptosporidium* by any rapid cartridge assay test. Samples came from community (hospital-based or reference) laboratories that had agreed to participate in the study with their State Public Health Laboratory. Eligible stool specimens were included in the study as they were reported, with a target of 100 samples per site, based on power calculations.

Stools were excluded from the study if 1) no clinical data were available, 2) no stool collection data were available, 3) there was insufficient (<5 g or ml) fecal material available for re-testing by both RCAs and DFA, or 4) the stool was stored in an unsuitable preservative (e.g., PVA fixatives).

### Data collection

Submitting community laboratories identified stools that met the study inclusion criteria and submitted samples to the respective public health laboratory. Specimen data that were collected by investigators from community laboratories included RCA test type, test lot number, test expiration date, sample preservative, specimen storage conditions, and specimen collection, receipt, and testing dates. Simple clinical and demographic data were collected by investigators at each of the study sites as part of routine disease surveillance activities based on the regulations of each site’s jurisdiction. Patients were interviewed using each state’s foodborne or *Cryptosporidium*-specific interview questionnaire to gather basic demographic data, including age, sex, clinical symptoms, onset and resolution dates, and hospitalizations. Interviews were conducted within 30 days of identifying the patient with laboratory-confirmed cryptosporidiosis.

Standardized data collection spreadsheets were developed and distributed by the CDC to the study sites. Data were collected on the submitting laboratory (community level) stool specimen and diagnostic assay, demographics gathered during interviews described above, and the test comparison completed at study sites. De-identified data was transmitted to the CDC for analyses.

### Laboratory methods

Testing was performed at the four participating state public health laboratories. The state public health laboratories performed the following tests concurrently on all stool specimens: 1) ImmunoCard STAT! Crypto/Giardia RCA by Meridian Bioscience; 2) Xpect *Cryptosporidium* RCA by Remel; and 3) Merifluor Crypto & Giardia DFA by Meridian Bioscience. Tests were stored, handled, and performed following manufacturers’ guidelines, including sample specifications such as storage condition and time from collection to testing. Additionally, molecular characterization was performed on specimens from Minnesota and Wisconsin. Minnesota conducted molecular testing at the Minnesota Department of Health Public Health Laboratory, and Wisconsin forwarded specimens to the CDC for molecular testing. Molecular testing at both sites included PCR-RFLP analysis of the 18s rRNA gene to differentiate *Cryptosporidium* species and genotypes and a nested amplification and sequencing of the gp60 gene to identify the subtype of *C*. *hominis*- and *C*. *parvum*-positive specimens [23].

### Data handling and analysis

Completed laboratory, specimen data, and case epidemiologic data were reviewed and coded by each site and were entered into a database created and supplied by CDC. Data analysis was performed at CDC using SAS 9.3 (Cary, NC). Log binomial regression was used to identify factors associated with false positive results at the community level while accounting for within-laboratory correlation.

## Results

A total of 386 stool specimens were submitted to the state public health laboratories (Minnesota, New York, and Wisconsin) by community laboratories. The Meridian ImmunoCard STAT!-positive specimens were the predominant submissions across all sites with 305 (79.0%) samples (Table 1). Age and gender were evenly represented in RCA positive, community submitted samples across all sites. Other characteristics of the specimens, including seasonality and preservative, were also evenly represented (Table 1).

**Table 1:** Characteristics of stool specimens that tested positive for *Cryptosporidium* infection by rapid cartridge assay at community laboratories, by study site and overall.

	Site			
	Minnesota	New York	Wisconsin	Overall
	N (%)	N (%)	N (%)	N (%)
**Total number of tests**	95 (24.6)	119 (30.8)	172 (44.6)	386 (100)
**Test**				
Meridian Immunocard Stat!	95 (100)	116 (97.5)	94 (54.7)	305 (79.0)
Remel Xpect	0	2 (1.7)	75 (43.6)	77 (20.0)
Other	0	1 (0.8)	3 (1.7)	4 (1.0)
**Age group**				
0–9 Years	14 (14.7)	22 (18.8)	49 (30.8)	85 (22.9)
10–19 Years	9 (9.5)	19 (16.2)	33 (20.8)	61 (16.4)
20–39 Years	30 (31.6)	34 (29.1)	37 (23.3)	101 (27.2)
40–59 Years	20 (21.1)	25 (21.4)	31 (19.5)	76 (20.5)
≥60 Years	22 (23.2)	17 (14.5)	9 (5.7)	48 (12.9)
**Sex**				
Male	49 (51.6)	47 (40.2)	83 (52.2)	179 (48.3)
Female	46 (48.4)	70 (59.8)	76 (47.8)	192 (51.8)
**Season**				
Summer*	46 (48.4)	66 (55.9)	90 (54.9)	202 (53.6)
Fall, Winter, Spring	49 (51.6)	52 (44.1)	74 (45.1)	175 (46.4)
**Storage temperature at community laboratory**				
Room temperature	87 (91.6)	99 (84.6)	88 (53.3)	274 (72.7)
Refrigerated (4C)	8 (8.4)	17 (14.5)	77 (46.7)	102 (27.1)
Other	0	1 (0.9)	0	1 (0.3)
**Preservative used**				
SAF	20 (21.1)	71 (59.7)	51 (29.7)	142 (36.8)
Cary Blair	30 (31.6)	0	81 (47.1)	111 (28.8)
10% Formalin	27 (28.4)	47 (39.5)	23 (13.4)	97 (25.1)
Other**	12 (12.6)	1 (0.8)	3 (1.7)	16 (4.2)
None	6 (6.3)	0	14 (8.1)	20 (5.2)
**Time between collection and testing at community laboratory**				
Same Day	61 (66.3)	46 (39.7)	102 (62.2)	209 (56.2)
Next Day	31 (33.7)	53 (45.7)	47 (28.7)	131 (35.2)
2–3 Days	0	17 (14.7)	15 (9.2)	32 (8.6)
**Confirmatory testing performed at community laboratory**				
Any	0	77 (65.3)	52 (30.8)	129 (34.9)
None	83 (100)	41 (34.8)	117 (69.2)	241 (65.1)

Percentages might not sum to 100 because of rounding. Total counts might not sum to 386 because of missing data.

* Summer was defined as sample receipt at the community laboratory between May 27, 2013 and September 30, 2013.

** “Other” category included Totalfix, Ecofix, and preservatives described as “Other” with no brand specified.

**Fig 1:**
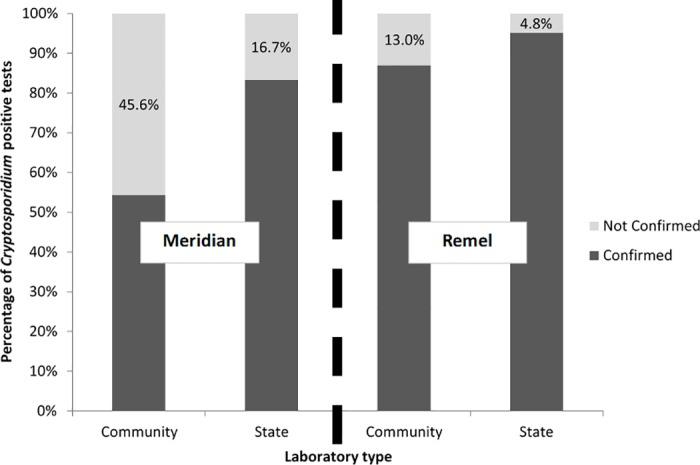
Comparison of positive predictive values for cryptosporidiosis rapid cartridge assay (RCA) tests performed in community laboratories and repeat testing under recommended conditions in state public health laboratories, by manufacturer.

Overall, 61.4% of community laboratory RCA-positive samples were confirmed by DFA; when RCA testing was repeated at the state laboratory, 86.4% of state laboratory RCA positives were confirmed by DFA (Table 2). The percentage of samples confirmed by DFA varied by manufacturer: 54.4% of the Meridian ImmunoCard STAT!-positives from community laboratories were confirmed by DFA and 87.0% of the Remel Xpect-positives from community laboratories could be confirmed. RCA positives were retested at the state laboratory, and 83.3% of Meridian ImmunoCard STAT! and 95.2% of Remel Xpect positives from state-administered tests were confirmed by DFA (Table 2, Fig 1). The proportion of community-tested specimens confirmed by DFA differed by patient age, ranging from 84.0% in persons <20 years of age to 12.5% in persons ≥60 years of age. That difference in concordant results was also found upon re-testing at the state laboratory.

**Table 2:** Results of direct fluorescent antibody testing (DFA) for stool specimens that tested positive for *Cryptosporidium* infection by rapid cartridge assay at community laboratories (n = 386) or repeat rapid cartridge assay at state public health laboratories (n = 249).

	Community laboratory		State laboratory	
	N positive	N (%) confirmed by DFA	N positive	N (%) confirmed by DFA
**Overall**	386	237 (61.4)	249	215 (86.4)
**Site**				
Minnesota	95	37 (39.0)	43	36 (83.7)
New York	119	76 (63.9)	81	70 (86.4)
Wisconsin	172	124 (72.1)	125	109 (87.2)
**Test**				
Meridian Immunocard! Stat	305	166 (54.4)	186	155 (83.3)
Remel Xpect	77	67 (87.0)	63	60 (95.2)
Other	4	4 (100.0)	NA	NA
**Age Group**				
0–9 Years	85	73 (85.9)	69	66 (95.7)
10–19 Years	61	53 (86.9)	47	46 (97.9)
20–39 Years	101	67 (66.3)	71	62 (87.3)
40–59 Years	76	34 (44.7)	40	32 (80.0)
≥60 Years	48	6 (12.5)	15	5 (33.3)
**Sex**				
Male	179	116 (64.8)	121	107 (88.4)
Female	192	117 (60.9)	122	104 (85.3)
**Season**				
Summer*	202	133 (65.8)	135	118 (87.4)
Fall, Winter, Spring	175	97 (55.4)	108	91 (84.3)
**Storage temperature at community laboratory**				
Room temperature	274	169 (61.7)	181	154 (85.1)
Refrigerated (4C)	102	63 (61.8)	62	56 (90.3)
Other	1	0 (0)	0	
**Preservative used**				
SAF	142	96 (67.6)	100	89 (89.0)
Cary Blair	111	58 (52.3)	70	53 (75.7)
10% Formalin	97	62 (63.9)	60	55 (91.7)
Other**	16	12 (75.0)	10	10 (100.0)
No preservative used	20	9 (45.0)	9	8 (88.9)
**Time between collection and testing at community laboratory**				
Same Day	209	114 (54.6)	122	102 (83.6)
Next Day	131	90 (68.7)	92	82 (89.1)
2–3 Days	32	23 (71.9)	26	22 (84.6)
**Confirmatory testing performed at community laboratory**				
Any***	129	97 (75.2)	101	88 (87.1)
None	241	134 (55.6)	141	122 (86.5)

* Summer was defined as sample receipt at the community laboratory between May 27, 2013 and September 30, 2013.

** “Other” category included Totalfix, Ecofix, and preservatives described as “Other” with no brand specified.

***”Any” category included DFA or IFA, staining and microscopy, and EIA

Because the majority of tests in this study were performed using the Meridian ImmunoCard STAT! kit, further analysis was done to understand predictors of false positivity for the test (Table 3). In univariate analysis, reporting state, patient age, and time between sample collection and testing at the community laboratory were significant predictors of false positive results; the type of preservative used approached significance. After adjusting for all other attributes, age and preservative type were significant predictors of false positive results.

**Table 3:** Predictors of false positive results (using direct fluorescent antibody testing (DFA) as the gold standard) for stool specimens that tested positive for Cryptosporidium infection by Meridian Immunocard Stat! rapid cartridge assay at community laboratories (n = 305).

	Total	Number (%) false positives	Unadjusted OR (95% CI)	Adjusted* OR (95% CI)
**Overall**	305	139 (45.6%)	NA	NA
**Site**				
New York	116	43 (37.1)	ref	ref
Wisconsin	94	38 (40.4)	1.2 (0.7, 1.9)	0.6 (0.3, 1.2)
Minnesota	95	58 (61.1)	2.7 (1.5, 4.7)	1.6 (1.0, 2.8)
**Age Group**				
≤25	140	31 (22.1)	ref	ref
>25	152	99 (65.1)	6.5 (3.6, 11.7)	6.3 (3.4, 11.7)**
**Sex**				
Male	142	60 (42.3)	ref	ref
Female	150	70 (46.7)	1.2 (0.8, 2.0)	0.8 (0.5, 1.5)
**Season**				
Summer	153	63 (41.2)	ref	ref
Fall, Winter, Spring	148	75 (50.7)	1.5 (0.9, 2.5)	1.7 (0.9, 3.2)
**Storage temperature at community laboratory**				
Room temperature	223	100 (44.8)	ref	ref
Refrigerated (4C)	74	35 (47.3)	1.1 (0.7, 1.9)	0.7 (0.3, 1.5)
**Preservative used**				
10% Formalin	90	35 (38.9)	ref	ref
SAF	100	43 (43.0)	1.2 (0.6, 2.4)	1.4 (0.9, 2.4)
Cary Blair	88	48 (54.6)	2.0 (1.0, 3.7)	3.0 (1.8, 4.9)**
Other***	15	4 (26.7)	0.6 (0.2, 1.3)	0.5 (0.4, 0.7)**
No preservative used	12	9 (75.0)	4.7 (1.0, 21.9)	8.1 (1.9, 35.4)**
**Time between collection and testing at community laboratory**				
Same Day	176	89 (50.6)	ref	ref
Next Day	99	38 (38.4)	0.6 (0.4, 0.9)	0.5 (0.3, 1.0)
2–3 Days	21	9 (42.9)	0.8 (0.3, 2.2)	0.8 (0.2, 2.9)

* Log binomial regression using GEE with an exchangeable correlation matrix was used to account for correlation between isolates from the same lab. The adjusted model included all attributes shown in the table.

** 95% confidence interval for odds ratios did not include the null after adjustment

*** “Other” category included Totalfix, Ecofix, and preservatives described as “Other” with no brand specified.

**Fig 2:**
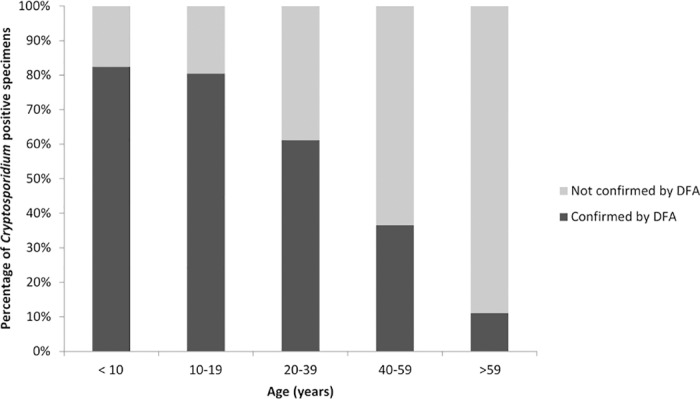
Age distribution of persons for which stool specimens tested positive for *Cryptosporidium* infection by Meridian ImmunoCard Stat! rapid cartridge assay at community laboratories, by DFA result.

A patient’s age was related to a higher false-positive rate of the Meridian ImmunoCard STAT!; those samples from individuals over age 25 were more likely to be unconfirmed between community and state testing (OR = 6.3 (3.4, 11.7)) (Table 3; Fig 2). Stool specimens stored in Cary Blair (OR = 3.0 (1.8, 4.9)) or without preservative (OR = 8.1 (1.9, 35.4)) and tested by the Meridican ImmunoCard STAT! were less likely to be confirmed between community and state laboratories, whereas those stored in “Other” preservatives (Totalfix, Ecofix, and no brand specified; OR = 0.5 (1.8, 4.9)) were more likely to be confirmed (Table 3). There were no significant differences in percentage of confirmed results based on sex, season, storage temperature, or time between collection and testing of samples (Table 3).

Molecular testing of Minnesota (n = 104) and Wisconsin (n = 180) stool specimens identified *C*. *parvum* (81.6%) and *C*. *hominis* (14.9%) as the predominant species; other species/genotypes identified included *C*. *ubiquitum* (n = 2), *C*. *meleagridis* (n = 1), and Cervine genotype (n = 1).

## Discussion

This study documented a high rate of false-positive results from RCAs used in community laboratories to test for *Cryptosporidium*. Nearly half of positive results from community laboratories could not be confirmed by DFA at the state public health laboratories. Because the sample was limited to positive results from community laboratories, a more thorough comparison of RCA and DFA at the state level could not be made, but the agreement between state DFA and RCA testing suggest that problems with test handling, storage, or operation in the community laboratories could account for at least a portion of the poor performance of RCAs. The high false-positive rate presents serious problems for routine surveillance and understanding the epidemiology of *Cryptosporidium* in the United States.

In this study, the performance of RCAs did not match manufacturer’s test performance evaluations. Several studies have previously identified these tests as having sensitivity and specificity similar to the performance of DFA, the gold standard test for *Cryptosporidium* [18–20]. However, previous problems with false-positive results associated with these tests have been noted [19, 24, 25]. These false positive issues were related to antigen production and delayed reading of results, which can lead to production of faint bands that could be misread as positives [22]. Similar concerns have not arisen from the commercially available antigens of traditional EIA tests.

Results suggest that factors in the community laboratory contributed to the decreased performance of the rapid cartridge assays. Possible contributors to decreased performance in community settings might include not reading the result at the prescribed time, inconsistency between test operators and readers, incorrect sample preservative, improper dilution of sample, use of out-of-date tests, and improper storage temperature of tests and/or specimens. These factors, especially testing times, could contribute to test performance errors, resulting in the appearance and misinterpretation of “ghost bands” on the RCA as a positive result. Education and training may help improve testing for *Cryptosporidium* in community settings (S1 Table). Possible areas for improvement include proper training on how to administer the test, better consistency in performance of the tests, and availability of simple guidelines for RCA procedures.

However, our data suggest that, even accounting for possible problems at community laboratories, these tests are performing at levels less than indicated in RCA package inserts that state 100% reproducibility. The positive predictive value at the state level during repeat testing suggests there are inherent specificity problems, particularly for the Meridian ImmunoCard STAT! RCA. Persons ≥65 years old were significantly more likely to have false positive results. This was true for tests performed at community and state public health laboratories, suggesting that this phenomenon is not due to poor testing practices. A possible explanation includes production of non-specific antibodies in the elderly that mimic *Cryptosporidium* epitopes. These mimic epitopes might react with the lateral flow assay to produce a false-positive result. False positives due to non-specific reactions in immunoassay tests have been previously reported, and decreased specificity among age groups have been noted in animal studies [24, 26]. Cross-reactivity of either RCA test’s proprietary antibodies with other organisms was not suspected as package inserts included a list of parasites, bacteria, and viruses tested for cross-reactivity during product development, quality assurance, and quality control. Another reason for the false positives may be inappropriate testing for cryptosporidiosis in the senior age group. Often, this population is less likely to have some of the major risk factors for acquiring *Cryptosporidium* infections, such as recreational swimming, coming in contact with children, and consumption of non-sterilized water [3]. Clinical manifestations are not pathognomonic for cryptosporidiosis; therefore, the common presentation of general gastroenteritis symptoms in seniors may lead to increased testing of the population and subsequent increased positive rate[27]. This finding is reflected in a recent analysis of cryptosporidiosis surveillance data which show that persons >65 years old have the second highest rate of illness [4]. If most of these cases are diagnosed by RCAs and there is a differential false positive rate for the elderly, these trends in national surveillance might be unreliable.

The problems with RCA use in community settings may decrease the utility of routine cryptosporidiosis surveillance data for understanding the *Cryptosporidium* epidemiology in the United States. Identifying factors contributing to decreased test performance are necessary to improve overall performance of these tests. Ideally, this will lead to greater confidence in the immunochromatographic card tests and expansion of the current CSTE classification criteria for confirmed cryptosporidiosis cases.

## Supporting Information

S1 Table Giardia and Cryptosporidium Rapid Cartridge Assay Guidelines for Laboratorians. (PDF)
