# Supplementary material for: Community Laboratory Testing for Cryptosporidium: Multicenter Study Retesting Public Health Surveillance Stool Samples Positive for Cryptosporidium by Rapid Cartridge Assay with Direct Fluorescent Antibody Testing
Source: PLoS One. 2017 Jan 13;12(1):e0169915. doi: 10.1371/journal.pone.0169915 (PMC5234809; doi:10.1371/journal.pone.0169915)
Supplement: S1 Table — (PDF) [file pone.0169915.s001.pdf]

Giardia and _Cryptosporidium_ Rapid Cartridge Assay Guidelines for Laboratorians

Remel x/pect *Giardia/Cryptosporidium* Rapid Test
Meridian Bioscience ImmunoCard STAT! *Crypto/Giardia* Rapid Assay

In recent years, investigations have determined that the rapid cartridge assays for simultaneous *Giardia* and *Cryptosporidium* analysis were resulting in false-positives. The positive bands may have been due to procedural problems. Because the problems were not associated with a particular lot, there has not been an FDA recall of these products. The following guidelines were developed to assist laboratorians in performing the rapid cartridge assays for simultaneous *Giardia* and *Cryptosporidium* analysis appropriately and to avoid false-positive results. These guidelines underscore potential problems with kit use. They do not differ from the manufacturers' written instructions.

Test Principle

The purpose of these qualitative immunochromatographic assays is to detect and distinguish *Giardia* and *Cryptosporidium* specific antigens in fecal specimens. Colored polystyrene particles or colloidal dye with labeled monoclonal antibodies to *Giardia* and *Cryptosporidium* is mixed with the fecal specimen and then dispensed onto the test cartridge. The cartridge contains a capture reagent or antibody for *Giardia*, a capture antibody for *Cryptosporidium*, and an antibody to bind excess colloidal dye or microparticles, which conjugates to form the control line. If *Giardia* or *Cryptosporidium* is present in the fecal specimen, a colored line (x/pect, blue for *Giardia,* red for *Cryptosporidium*; STAT!, gray-black for both) will be present in their respective windows in addition to a line in the control window.

Positive and Negative Controls

We recommend the use of external positive and negative controls for quality assurance purposes. The Meridian Merifluor DFA Kit for *Cryptosporidium/Giardia* or examination of modified Kinyoun's acid-fast stained smears for *Cryptosporidium* spp. or Wheatley's trichrome stain for *Giardia* spp. are reliable confirmatory methods for the identification of these organisms.

Procedural Guidelines

	Remel Xpect *Giardia/Cryptosporidium* Rapid Test	Meridian Bioscience ImmunoCard STAT! *Crypto/Giardia* Rapid Assay
Specimen Collection	Specimens should be collected in **10% Formalin, SAF, MIF, Cary-Blair, or Stuart's (STAT! only) media.** If the fecal specimen is **fresh**, the sample must be **diluted 1:4** in deionized water or one of the above mentioned transport media before testing. Specimens in PVA or concentrated specimens should NOT be used. Ecofix and UniFix have not been validated for use with these kits; the manufacturer of TotalFix indicates compatibility. Concentrated samples should not be used because the antigen is primarily soluble.	
Sample Volume	The transfer pipet included with the kit draws approximately 100 µl. If not using the included pipet, be sure to add approximately 100 µl of the specimen. Use the liquid fraction of the specimen, avoiding large insoluble debris.	The transfer pipet included with the kit draws approximately 60 µl. If not using the included pipet, be sure to add approximately 60 µl of the specimen. Use the liquid fraction of the specimen, avoiding large insoluble debris.
Reagent Temperature/Storage	Store the kit **refrigerated** at 2-8°C. DO NOT freeze. Refrigerated kits must come to room temperature before use. Do not remove test from pouch until ready for use. When finished, **promptly** return the kit to the refrigerator. Reagents from different lots should not be interchanged.	
Sample Temperature/Storage	Samples must be brought to room temperature before testing. **Formalin, SAF, and MIF** samples that are kept at room temperature, refrigerated or frozen should be tested within **2 months** of collection. **Cary Blair** samples must be **refrigerated and tested within 1 (x/pect) to 2 weeks (STAT!)** of collection or kept **frozen and tested within 2 months**. **Stuart's medium (STAT! only) or fresh stool must be tested as soon as possible**. Avoid multiple freeze-thaw cycles.	
Reading Results	Read the results window at **15 minutes**. **Results are invalid beyond 15 minutes**. Visible test lines of any intensity of blue and/or red to pink should be read as positive. The control line must be present or the test is invalid. An incomplete line in any position is an invalid test.	Read the results window after **10 minutes but before 15 minutes**. **Results are invalid after 15 minutes**. Visible black test lines of any intensity should be read as positive. The control line must be present or the test is invalid. An incomplete or beaded line in any position is an invalid test.
Quality Control	Quality control samples should be run with each new kit lot. Known positive and negative formalin-preserved specimens should be used.	
Detection limits	Negative results may occur in samples containing levels of antigen below the detection limit of the assay.	
